# Pharmacists’ Utilization of Information Sources Related to Community and Population Needs in the Upper Midwest and Associations with Continuing Professional Education

**DOI:** 10.3390/pharmacy7030125

**Published:** 2019-08-29

**Authors:** Paul Henkel, Marketa Marvanova

**Affiliations:** 1 Department of Geographical and Historical Studies, University of Eastern Finland, P.O. Box 111, FI-80101 Joensuu, Finland; 2 Department of Pharmacy Practice, School of Pharmacy/College of Health Professions, North Dakota State University, Department 2650, PO Box 6050, Fargo, ND 58108-6050, USA

**Keywords:** pharmacist, population needs, information, continuing education, pharmacy workforce, rural communities

## Abstract

Background: To investigate information sources utilized in pharmacists’ assessment of population-based health needs and/or community changes; and the association between information sources utilized and reported completion of continuing professional education topics. Methods: In 2017; licensed pharmacists (n = 1124) in North Dakota; South Dakota; Minnesota; Iowa; and Nebraska completed a questionnaire on continuing professional education and information sources on population-based health needs and community changes. Data were entered; cleaned and imported into Stata 11.1. Census Bureau county-level population density data were used to classify local area characteristics. Descriptive statistics and multivariate logistic regression analyses were performed. Results: Most sources of primary; county-level data on population-based health needs or community changes were minimally utilized. Pharmacists in more rural areas were statistically more likely to use local health professionals; local non-health professionals; and/or the state health department compared to pharmacists in less rural areas. Pharmacists reporting higher use of population-based information sources were more likely to have completed continuing education in the past 12 months for all 21 surveyed topics; 13 significantly so. Conclusions: There is a reliance of pharmacists on information from local health and non-health professionals for information on population-based health needs and/or community changes. Utilization of health departments and other primary information sources was associated with increased rates of completion of an array of continuing professional education topics. Expanding utilization of evidence-driven information sources would improve pharmacists’ ability to better identify and respond to population-based health needs and/or community changes through programs and services offered; and tailor continuing professional education to population-based health needs.

## 1. Introduction

In the United States (U.S.), pharmacists are one of the most accessible health professionals and represent a significant component of the trained healthcare workforce in both urban and rural communities [1,2]. The landscape of public health and roles of pharmacists are changing as a result of continuing professional education (CPE) practices as well as due to changes in population-based health needs and healthcare delivery across the U.S. [3,4,5] While traditional pharmacy services in community pharmacies, hospitals, and long-term care facilities persist, there are new pharmacy services and pharmacists’ roles that extend beyond medication dispensing and distribution. Pharmacists are currently integrated into public health functions, specifically in health promotion, prevention, and health/medication information such as preventative health screenings [4,6,7,8,9,10,11]. Pharmacists working in all pharmacy practice settings should ensure appropriate incorporation of public health concepts, issues, information, and services into their practice. Although there is a general understanding of current public health services provided by pharmacists, especially in community pharmacies, there is limited information about specific resources utilized by pharmacists to self-educate about population-based health needs and community changes.

In providing or considering provision of health promotion services, it is essential that healthcare professionals utilize up-to-date, evidence-based information to understand and attempt to address population-based health needs and community changes [1,12]. Access to quality, locally-relevant, evidence-based information has been recognized for decades as being requisite for high-functioning multidisciplinary and inter-professional teams pursuing effective, integrated, population-based outreach and interventions [13,14]. The ability to identify and obtain information that is relevant for a local community or market area and to then effectively utilize that information in pharmacy practice is crucial [15]. For rural healthcare practitioners in particular (defined as pharmacists practicing in areas with 25 or fewer persons per square mile, see methods for a full description), obtaining information that is high-quality but relevant to a local area can be a significant and challenging barrier, since information is sometimes available at the county-level, but often only for larger area aggregates [16].

All communities are facing changes: lack of health insurance, health professional shortages, longer distances to obtain care, social stigma/privacy concerns, and poor health literacy are all significant in accessing and obtaining care [17,18,19]. One of the most significant changes impacting healthcare in recent decades has been the growing U.S. aging population, both in absolute number and as a percent of the population, a demographic shift which has impacted many rural areas [18,20,21]. Many pharmacists are well-positioned to serve population-based health needs of local residents. One way to do so is by expanding services for public health, but there currently exist significant obstacles in doing so [6,22]. 

In providing pharmacy-related services to a community, locally- and population-relevant information can aid in: measuring burden or understanding changes in disease/needs/prevention, guiding and planning product delivery, program/business development, services provision, evaluating national and state policy impacts on local communities/populations, prioritizing financial and human capital, evaluating effectiveness, and balancing effectiveness with efficiency [23]. However, an equally important function of this information (or an understanding of the lack thereof) is ensuring that the pharmacist has the necessary knowledge and skills to meet those population-based health needs or community changes. Given the limited amount of prior research on CPE practices and information resource utilization among pharmacists, along with poor understanding of how pharmacists obtain information about local population-based health needs and community changes, we had two study objectives: 

Firstly, to understand pharmacists’ utilization of information sources to identify their needs related to population-based needs and community changes, specifically whether there were systematic differences in the types of resources used by practice setting. Secondly, to investigate the association between information sources utilized and reported completion of surveyed CPE topics, primarily in geriatric care and diseases of public health importance, in the past 12 months.

## 2. Materials and Methods 

Between March and June 2017, licensed, resident pharmacists in five states: Iowa (IA), Minnesota (MN), Nebraska (NE), North Dakota (ND), and South Dakota (SD) were surveyed on information sources they had utilized in the past 12 months to ascertain population-based health needs and/or community changes. This study was approved by the University’s Institutional Review Board and informed consent was obtained from eligible participants. In this cross-sectional study, all pharmacists in IA, NE, ND, and SD were invited to complete the questionnaire, while a geographically-stratified (county-level) sample of pharmacists in MN were invited to complete the questionnaire, with response rates ranging from 9.7–17.3% [24].

Pharmacists were surveyed about professional, educational, personal characteristics, and workplace, and were asked in which topics they had completed any CPE hours in the past 12 months [24]. They were also asked which sources of information they had utilized in the past 12 months to understand population-based health needs and/or community changes. Sources were derived from several public health resource pages on primary population/community-based information, pharmacy education standards, and from the authors’ prior research. Sources included: local community leaders (non-health), local health professionals, city government, county government, state government, professional organization (s), Centers for Disease Control and Prevention (CDC), Medicare.gov, Agency for Healthcare Research and Quality (AHRQ), American FactFinder, Behavioral Risk Factor Surveillance System (BRFSS), CDC Wonder, Dartmouth Atlas, National Center for Health Statistics (NCHS), National Health and Nutrition Examination Survey (NHANES), and U.S. Census Bureau. Participants were also invited to enter any other sources of information utilized in a text-free field.

Respondents (n = 1239) completed either a web-based questionnaire (hosted by the University, using Qualtrics) or a hardcopy questionnaire (including pre-paid return envelope), the latter were collected and entered into the Qualtrics database by the researchers using the web-based form. Data were downloaded, imported into Microsoft Excel, cleaned, and coded. Population and geographical area data were obtained from the United States Census Bureau’s American Community Survey 5-Year Estimates [25], cleaned, and merged with the study data set. Respondents (n = 115) for whom there was not a county or zip code identifier were excluded from the analysis. Rural areas (19.9%) were defined using two Census variables (population, land area) as those with 25 persons or fewer per square mile. Less rural areas were categorized using distribution into 25.1–100.0 persons per square mile (35.1%), 100.1–300.0 persons per square mile (17.8%), and >300.0 persons per square mile (27.2%), generally corresponding to rural, small/large town, small city, large city classifications regionally. Descriptive and logistic regression analyses were performed using Stata 11.1. 

## 3. Results

A total of 1124 respondents were included in the analysis (Table 1). The majority of pharmacists were female (65.6%), had completed a Doctor of Pharmacy degree (61.4%), had ≥10 years of experience (70.5%), were not Board of Pharmacy Specialties (BPS)-certified (86.4%), and had not completed residency training (81.4%). Most pharmacists worked in community pharmacies (46.0%), hospitals (27.8%), clinics (7.2%), and long-term care (5.4%). Others (11.1%) were retired or reported a primary workplace in government, home-infusion, pharmaceutical company/industry, compounding, specialty pharmacy, remote-order entry, sterile compounding, mail-order pharmacy or hospital/government administration (Table 1).

After adjusting for terminal pharmacy degree, pharmacy residency, BPS certification, and workplace area population density, there were few differences between respondents working in community pharmacies compared to those practicing in hospitals or clinics, excepting that those practicing in hospitals or clinics were less likely to report seeking information on population-based health needs and/or community changes using Medicare compared to those in community settings (Table 2). Respondents practicing in hospitals and clinics were also more likely to report seeking information using professional organizations. Primary information sources such as American FactFinder, CDC Wonder, NHANES, BRFSS, and Dartmouth Atlas were used by few respondents overall but included respondents from all pharmacy workplace settings.

Pharmacists reported using an average of 1.97 ± 1.77 information sources on population-based health needs and/or community changes (Table 2). After adjusting for terminal pharmacy degree, pharmacy residency, BPS certification, and workplace type, the respondents in the more rural areas (≤25.0 persons/square mile or 25.1–100.0 persons/square mile) were significantly more likely to report seeking information from local health professionals, local non-health professionals, and/or the state health department compared to pharmacists in less rural areas. It should be noted that pharmacists in the most rural areas (≤25.0 persons/square mile) were the most likely to report utilizing the state health department for information on population-based health needs and/or community changes, but only a scant 8.5% of those rural pharmacist respondents reported utilizing this information source in the past 12 months (Table 3).

Both community pharmacists (see Table 4) and hospital pharmacists (see Table 5) in the most rural areas (≤25.0 persons/square mile) were significantly more likely to report using local health professionals, local non-health professionals, and state health departments compared to their peers in less rural areas. Community pharmacists in areas with ≤25.0 persons/square mile were also more likely to report seeking information on population-based health needs and/or community changes from professional associations compared to their peers in less rural areas (Table 4). Hospital pharmacists in the most rural areas were significantly more likely to report using the CDC website when seeking information on population-based health needs and/or community changes compared to their peers in less rural areas (Table 5).

Utilization of evidence-based information sources on population-based health needs and/community changes was strongly associated with completion rates of selected continuing education topics. Of the 21 topics surveyed, pharmacists who reported using one or more evidence-based information sources (beyond local health professionals, local non-health professionals, and/or professional associations) reported higher rates of completion of hours in each topic. These associations were statistically significant at the *p* < 0.05 level or lower for 13 of the 21 topics. (See Table 6).

## 4. Discussion

Pharmacists surveyed in the Upper Midwest (IA, NE, ND, MN, SD) reported utilization of relatively few information sources (mean = 1.97 ± 1.77) in the past 12 months. Pharmacists were specifically asked about the information sources utilized for “population needs and/or changes in your community.” Sources reported as used by at least one-fourth of pharmacists, in descending order, were: local health professionals (47.7%), CDC website (37.2%) and professional associations (34.5%). While local health professionals do possess information about local needs and community changes, this should not be considered a quality source for evidence-based information but rather anecdotal information, not the best “go to” source for information unless no other evidence-based information is available. The CDC website is highly evidence-based, but information is predominantly available on disease conditions and trends, with the vast majority of data presented at the national- or state-level [26]. This provides general patterns and trends for the U.S., but is quite an ineffectual resource for persons seeking city- or county-specific information on population-based health needs and community changes. Professional associations, either at the state- or national-level, provide information, but national-level associations provide little to nothing on local population-based health needs and community changes, excepting presentations at meetings by individuals about their own communities. State-level associations provide information that might have more local relevance, and both national and state associations are likely to offer webinars and/or continuing pharmacy education that have some bearing on issues faced at the local level. Far more often than not, these are not specific as to population-based health needs and community changes for multiple localities. The primary Medicare website was also heavily utilized but predominantly provides information for consumers and providers about administration, eligibility, and reimbursement for Medicare programs, with relatively little provided about population-based health needs and community changes at the local or even regional levels [27].

Overall, 16.7% of pharmacists reported utilizing the city or county health department as an information source on population-based health needs or community changes, and only 1 in 20 pharmacists had utilized the state health department. State, county, and city health departments work to serve statewide and local health needs, typically providing extensive data at least annually and, as public agencies, will work with community partners and organizations to serve data and information needs related to population-based health needs and community changes. Only 1 in 6 pharmacists reported using any health department in the past 12 months, a rate which is particularly noteworthy when this is among the best of resources for rural areas or for a specific locality within a state. Almost no pharmacists utilized any of the specialized, web-based, free-to-use primary information resources available from national agencies and organizations which do allow for data to be refined to county-level (typical) or local area (possible). While this was not a surprise, these websites have become much more user friendly in the past 10 years, and may be underutilized because pharmacists simply do not know of their existence, or have used them in the past but previously found them too difficult to use for obtaining information on population-based health needs or community changes.

Community pharmacists, as suspected, were more likely to utilize the Medicare website compared to peers practicing in hospitals and clinics. The Medicare website provides extensive information on administration, billing, and reimbursement, but very little on information related to population-based health needs or community changes related to outcomes [27]. Community pharmacists were no more likely to utilize city, county, and/or state health departments as an information source compared to pharmacists from any other pharmacy practice setting. Pharmacists in academia were far more likely to report having utilized primary evidence-based information sources like the Census Bureau and ARHQ.

Surveyed pharmacists reported proportionately high rates of utilization of local health professionals to inform themselves on population-based health needs and community changes. However, compared to pharmacists in more urban areas, pharmacists in the most rural areas reported a significantly greater reliance on local health and local non-health professionals. Pharmacists in all but the most urban areas (≥300.0 persons per square mile) were significantly more likely to report utilizing the health department, but only 5.0–8.5 percent of pharmacists in more rural areas reported utilizing this information source for population-based health needs and community changes in the past 12 months. While local health and non-health professionals are valuable sources of qualitative and anecdotal information, this type of information is not a viable substitute for scientifically collected, prepared, and released data on population-based health needs and community changes such as is available from health departments. The latter type of information sources provides data of the highest value for provision of quality, evidence-based care and for development of new or improved services to meet local health needs.

For community pharmacists, information source utilization based on local area population density, as rurality increased, so did reliance on local health professionals and local non-health professionals. Only modest increases were observed for utilization of the state health department. No differences were reported in city or county health departments based on rurality. Fewer than 5 percent of community pharmacists overall and only 6.4 percent of community pharmacists in the most rural areas (≤25.0 persons per square mile) reported utilizing the state health department in the past 12 months. Based on utilization rates alone, community pharmacists were far more likely to utilize local health and non-health professionals compared to state or county/city health departments. Hospital pharmacists’ utilization of information sources, based on rurality, increased significantly for the most rural areas (≤25.0 persons/square mile) with respect to local health professionals, the CDC website, local non-health professionals, and the state health department, with no such differences observed for areas with 25.1–100.0 persons/square mile compared to more urban areas. Despite this, the state health department was only utilized by 10.4% of hospital pharmacists in the past 12 months. Irrespective of pharmacy practice setting (community vs. hospital), evidence-based sources of information are seemingly not the predominating sources of information utilized by pharmacists on population-based health needs and community changes. Given the nature of anecdotal information, this creates severe limitations when it comes to meeting local current and future health needs through innovative products, services, and programs.

Pharmacists utilized population-based information sources at significantly lower rates than the other more commonly utilized, anecdotal information sources. Knowing this, utilization of population-based resources was strongly associated with likelihood of completion of CPE topics in a diverse array of areas. Pharmacists’ utilization of population-based information resources was associated in higher rates of completion of hour(s) in CPE topics (in the past 12 months) in all of the 21 topics surveyed, statistically significantly for 13 of 21 topics, including but not limited to: Alzheimer disease, Chronic Obstructive Pulmonary Disease, Diabetes, Health Disparities, Hypertension, and Parkinson disease. While we cannot ascertain whether the information utilization practices drove continuing professional topic selection, or whether there is another construct or factor related to both, what is clear is that there is a correlation between information-seeking practices on population-based health needs and community changes, and the selection and completion of CPE topics related to these needs and changes.

This study comprised licensed pharmacists from only 5 states in the Upper Midwest (IA, NE, ND, MN, SD). While findings were quite consistent among surveyed states, we understand that it may not be possible to extrapolate these findings to other geographic areas. The study was also cross-sectional in design and relied solely upon pharmacists’ self-reporting of information source utilization and CPE completion in the past 12 months. 

## 5. Conclusions

This study revealed that among surveyed pharmacists practicing in IA, MN, NE, ND, and SD, there were very low reported rates of utilization of primary, evidence-based information sources on population-based health needs and/or community changes, irrespective on pharmacy practice setting or location. For serving the public’s health and for providing quality services that meet needs of changing local populations, primary, reliable, population-based information is essential for evidence-based pharmacy practice. There is value in and importance of information gathered from local health and non-health professionals, however this information is not a quality substitute for scientifically collected, analyzed, and verified information essential for informing pharmacists on population-based health needs or community changes in a local area. There is a need for broadening knowledge on and expanding utilization of evidence-driven information sources among pharmacists to better serve in public health role(s) and better identify and respond to population-based health needs and/or community changes through program and service offerings. In concordance, Center for the Advancement of Pharmaceutical Education (CAPE) Outcomes and American Association of Colleges of Pharmacy (AACP) statements require priorities and goals for pharmacists’ education that would not only be patient-centered, but also population-driven, evidence-based, and public health grounded [28]. Therefore, Schools and Colleges of Pharmacy should ensure that new graduates possess the knowledge, skills and abilities to ensure the current pharmacy workforce is ready to serve in population health roles. For those individuals who are already in the pharmacy workforce, continuous professional development opportunities are needed which include training in identifying, accessing, and utilization of high-quality, population-based information sources.

## Figures and Tables

**Table 1:** Respondents Demographics (n = 1239).

	Number of Persons Per Square Mile				
	>300.0	100.1–300	25.1–100.0	≤25.0	ALL
	n (%)	n (%)	n (%)	n (%)	n (%)
SEX					
Male	95 (31.1)	66 (33.0)	143 (36.2)	83 (37.2)	387 (34.4)
Female	211 (68.9)	134 (67.0)	252 (63.8)	140 (62.8)	737 (65.6)
**TERMINAL PHARMACY DEGREE**					
B.S.	114 (37.4)	60 (30.3)	154 (39.6)	102 (46.0)	430 (38.6)
Pharm.D.	191 (62.6)	138 (69.7)	235 (60.4)	120 (54.0)	684 (61.4)
**PHARMACY EXPERIENCE**					
<10 Years	76 (24.8)	66 (33.2)	121 (30.9)	68 (36.4)	331 (29.5)
≥10 Years	230 (75.2)	133 (66.8)	271 (69.1)	156 (69.6)	790 (70.5)
**COMPLETED PHARMACY RESIDENCY**					
No	245 (80.1)	129 (65.5)	328 (84.5)	203 (91.9)	905 (81.4)
Yes	61 (19.9)	68 (34.5)	60 (15.5)	18 (8.1)	207 (18.6)
**BPS CERTIFICATION ^a^**					
No	254 (83.0)	150 (75.0)	359 (90.9)	209 (93.3)	972 (86.4)
Yes	52 (17.0)	50 (25.0)	36 (9.1)	15 (6.7)	153 (13.6)
**PHARMACY WORKPLACE**					
Community	122 (40.0)	57 (28.5)	196 (50.0)	140 (63.1)	515 (46.0)
Hospital	80 (26.2)	71 (35.5)	112 (28.6)	48 (21.6)	311 (27.8)
Clinic	16 (5.3)	26 (13.0)	26 (6.6)	13 (5.9)	81 (7.2)
LTC	18 (5.9)	12 (6.0)	26 (6.6)	4 (1.8)	60 (5.4)
Academia/Research	14 (4.6)	11 (5.5)	3 (0.8)	0 (0.0)	28 (2.5)
Other ^b^	55 (18.0)	23 (11.5)	29 (7.4)	17 (7.7)	124 (11.1)
**PRECEPTOR**					
No	115 (37.7)	104 (52.5)	131 (33.3)	89 (39.9)	439 (39.2)
Yes	190 (62.3)	94 (47.5)	262 (66.7)	134 (60.1)	680 (60.8)

Abbreviations: Bachelor of Science (B.S.); Doctor of Pharmacy (Pharm. D.); Board of Pharmacy Specialties (BPS); Long term care (LTC); standard deviation (SD). ^a^ BCACP = Board Certified Ambulatory Care Pharmacist, BCGP = Board Certified Geriatric Pharmacist, BCPP = Board Certified Psychiatric Pharmacists, BCPS = Board Certified Pharmacotherapy Specialist. ^b^ Other includes: retired, government, home-infusion, pharmaceutical company/industry, compounding, specialty pharmacy, remote-order entry, sterile compounding, mail-order pharmacy or hospital/government administration.

**Table 2:** Information Source Utilization by Pharmacists’ Workplace Type (n = 1239).

Information Source	Workplace						
	Community	Hospital	Clinic	LTC	Academia	Other ^a^	ALL
Number Utilized; mean ± SD	1.91 ± 1.61	1.93 ± 1.72	2.13 ± 1.81	2.15 ± 1.86	3.09 ± 2.86	1.90 ± 1.95	1.97 ± 1.77
Local Health Professionals; n (%)	276 (49.1)	179 (50.9)	49 (51.0)	36 (50.7)	13 (40.6)	57 (34.3)	610 (47.7)
CDC Website; n (%)	177 (31.5)	153 (43.5)	38 (39.6)	26 (36.6)	17 (53.1)	**65 (39.2)**	476 (37.2)
Professional organizations; n (%)	159 (28.3)	128 (36.4)	**45 (46.9)**	27 (38.0)	**20 (62.5)**	62 (37.4)	441 (34.5)
Medicare; n (%)	173 (30.8)	**49 (13.9)**	**22 (22.9)**	27 (38.0)	9 (28.1)	29 (17.5)	309 (24.2)
Local Non-Health Professionals; n (%)	129 (23.0)	59 (16.8)	14 (14.6)	9 (12.7)	7 (21.9)	26 (15.7)	244 (19.1)
City/County Health Department n (%)	99 (17.6)	48 (13.6)	15 (15.6)	10 (14.1)	6 (18.8)	35 (21.1)	213 (16.7)
AHRQ; n (%)	8 (1.4)	21 (6.0)	7 (7.3)	**8 (11.3)**	**9 (28.1)**	**13 (7.8)**	66 (5.2)
State Health Department; n (%)	27 (4.8)	15 (4.3)	7 (7.3)	4 (5.6)	0 (0.0)	**11 (6.6)**	64 (5.0)
Census Bureau; n (%)	11 (2.0)	11 (3.1)	2 (2.1)	1 (1.4)	**5 (15.6)**	7 (4.2)	37 (2.9)
NCHS; n (%)	4 (0.7)	10 (2.8)	1 (1.0)	1 (1.4)	4 (12.5)	6 (3.6)	26 (2.0)
CDC Wonder; n (%)	5 (0.9)	4 (1.1)	2 (2.1)	2 (2.8)	2 (6.3)	1 (0.60)	16 (1.3)
BRFSS; n (%)	1 (0.2)	1 (0.3)	2 (2.1)	1 (1.4)	4 (12.5)	2 (1.2)	11 (0.9)
NHANES; n (%)	2 (0.4)	1 (0.3)	0 (0.0)	1 (1.4)	2 (6.3)	1 (0.6)	7 (0.6)
American Fact Finder; n (%)	1 (0.2)	0 (0.0)	0 (0.0)	0 (0.0)	1 (3.1)	0 (0.0)	2 (0.2)
Dartmouth Atlas; n (%)	0 (0.0)	0 (0.0)	0 (0.0)	0 (0.0)	0 (0.0)	1 (0.6)	1 (0.1)

Abbreviations: Agency for Healthcare Research and Quality (AHRQ); Behavioral Risk Factor Surveillance System (BRFSS); Centers for Disease Control and Prevention (CDC); confidence interval (CI); National Center Health Statistics (NCHS); National Health and Nutrition Examination Survey (NHANES); long term care (LTC); odds ratio (OR); standard deviation (SD). ^a^ Other includes: government, home-infusion, pharmaceutical company/industry, compounding, specialty pharmacy, remote-order entry, sterile compounding, mail-order pharmacy or hospital/government administration Statistically significant values (*p* < 0.05) are in bold text, calculating using logistic regression analysis adjusted for terminal pharmacy degree, pharmacy residency, Board of Pharmacy Specialties certification, and population density are in bold text.

**Table 3:** Information Source Utilization by Pharmacists’ Workplace’s Local Area Population Density (n = 1239).

Information Source	Number of Persons Per Square Mile				
	≥300.0	100.1–300	25.1–100.0	≤25.0	ALL
Sources Utilized; mean ± SD	1.95 ± 1.81	1.95 ± 1.79	1.92 ± 1.69	2.18 ± 1.81	1.97 ± 1.77
Local Health Professionals; n (%)	126 (41.2)	87 (43.5)	**206 (52.2)**	**122 (54.5)**	541 (48.1)
CDC Website; n (%)	123 (40.2)	80 (40.0)	138 (34.9)	78 (34.8)	419 (37.2)
Professional organizations; n (%)	112 (36.6)	82 (41.0)	114 (28.9)	83 (37.1)	391 (34.8)
Medicare; n (%)	85 (27.8)	**38 (19.0)**	96 (24.3)	58 (25.9)	277 (24.6)
Local Non-Health Professionals; n (%)	46 (15.0)	28 (14.0)	**83 (21.0)**	**65 (29.0)**	222 (19.7)
City/County Health Department; n (%)	42 (13.7)	25 (12.5)	48 (12.2)	31 (13.8)	146 (13.0)
AHRQ; n (%)	26 (8.5)	**12 (6.0)**	14 (3.5)	5 (2.2)	57 (5.1)
State Health Department; n (%)	3 (1.0)	**10 (5.0)**	**21 (5.3)**	**19 (8.5)**	53 (4.7)
Census; n (%)	11 (3.6)	6 (3.0)	9 (2.3)	8 (3.6)	34 (3.0)
NCHS; n (%)	9 (2.9)	6 (3.0)	6 (1.5)	2 (0.9)	23 (2.0)
CDC Wonder; n (%)	2 (0.7)	3 (1.5)	5 (1.3)	2 (0.9)	12 (1.1)
BRFSS; n (%)	6 (2.0)	2 (1.0)	2 (0.5)	1 (0.5)	11 (1.0)
NHANES; n (%); n (%)	4 (1.3)	2 (1.0)	1 (0.3)	0 (0.0)	7 (0.6)
Fact Finder; n (%); n (%)	1 (0.3)	0 (0.0)	0 (0.0)	1 (0.5)	2 (0.2)
Dartmouth Atlas; n (%)	0 (0.0)	0 (0.0)	1 (0.3)	0 (0.0)	1 (0.1)

Abbreviations: Agency for Healthcare Research and Quality (AHRQ); Behavioral Risk Factor Surveillance System (BRFSS); Centers for Disease Control and Prevention (CDC); confidence interval (CI); National Center Health Statistics (NCHS); National Health and Nutrition Examination Survey (NHANES); odds ratio (OR); standard deviation (SD). Statistically significant values (*p* < 0.05) are in bold text, calculating using logistic regression analysis adjusted for terminal pharmacy degree, pharmacy residency, Board of Pharmacy Specialties certification, and population density.

**Table 4:** Information Source Utilization by Community Pharmacists by Local Area Population Density (n = 562).

Information Source	Number of Persons Per Square Mile				
	≥300.0	100.1–300	25.1–100.0	≤25.0	ALL
Sources Utilized; mean ± SD	1.72 ± 1.42	1.88 ± 1.58	1.89 ± 1.60	2.06 ± 1.77	1.91 ± 1.61
Local Health Professionals; n (%)	49 (40.2)	22 (38.6)	**103 (52.6)**	**75 (53.6)**	249 (48.4)
CDC Website; n (%)	41 (33.6)	22 (38.6)	61 (31.1)	35 (25.0)	159 (30.9)
Professional organizations; n (%)	31 (25.4)	17 (29.8)	46 (23.5)	**52 (37.1)**	146 (28.4)
Medicare; n (%)	44 (36.1)	18 (31.6)	60 (30.6)	42 (30.0)	164 (31.8)
Local Non-Health Professionals; n (%)	16 (13.1)	10 (17.5)	**47 (24.0)**	**44 (31.4)**	117 (22.7)
City/County Health Department; n (%)	17 (13.9)	9 (15.8)	27 (13.8)	19 (13.6)	72 (14.0)
AHRQ; n (%)	3 (2.5)	2 (3.5)	2 (1.0)	0 (0.0)	7 (1.4)
State Health Department	1 (0.8)	3 (5.3)	9 (4.6)	**9 (6.4)**	22 (4.3)
Census; n (%)	3 (2.5)	1 (1.8)	4 (2.0)	3 (2.1)	11 (2.1)
NCHS; n (%)	2 (2.5)	0 (0.0)	0 (0.0)	1 (0.7)	4 (0.8)
CDC Wonder; n (%)	0 (0.0)	1 (1.8)	2 (1.0)	1 (0.7)	4 (0.8)
BRFSS; n (%)	0 (0.0)	0 (0.0)	0 (0.0)	1 (0.7)	1 (0.2)
NHANES; n (%)	1 (1.8)	0 (0.0)	1 (0.5)	0 (0.0)	2 (0.4)
Fact Finder; n (%)	0 (0.0)	0 (0.0)	0 (0.0)	1 (0.7)	1 (0.2)
Dartmouth Atlas; n (%)	0 (0.0)	0 (0.0)	0 (0.0)	0 (0.0)	0 (0.0)

Abbreviations: Agency for Healthcare Research and Quality (AHRQ); Behavioral Risk Factor Surveillance System (BRFSS); Centers for Disease Control and Prevention (CDC); confidence interval (CI); National Center Health Statistics (NCHS); National Health and Nutrition Examination Survey (NHANES); odds ratio (OR); standard deviation (SD).Statistically significant values (*p* < 0.05) are in bold text, calculating using logistic regression analysis adjusted for terminal pharmacy degree, pharmacy residency, Board of Pharmacy Specialties certification, and population density.

**Table 5:** Information Source Utilization by Hospital Pharmacists by Local Area Population Density (n = 352).

Information Source.	Number of Persons Per Square Mile				
	≥300.0	100.1–300	25.1–100.0	≤25.0	ALL
Sources Utilized; mean ± SD	1.84 ± 1.65	1.82 ± 1.76	1.74 ± 1.56	2.71 ± 1.97	1.93 ± 1.72
Local Health Professionals; n (%)	37 (46.3)	32 (45.1)	58 (51.8)	**32 (66.7)**	159 (51.1)
CDC Website n (%)	34 (42.5)	30 (42.3)	40 (35.7)	**29 (60.4)**	133 (42.8)
Professional organizations n (%)	32 (40.0)	32 (45.1)	34 (10.4)	18 (37.5)	116 (37.3)
Medicare n (%)	10 (12.5)	7 (9.9)	15 (13.4)	9 (18.8)	41 (13.2)
Local Non-Health Professionals n (%)	12 (15.0)	7 (9.9)	21 (18.8)	**15 (31.3)**	55 (17.7)
City/County Health Department n (%)	10 (12.5)	6 (8.5)	10 (8.9)	8 (16.7)	34 (10.9)
AHRQ n (%)	5 (6.3)	5 (7.0)	5 (4.5)	4 (8.3)	19 (6.1)
State Health Department n (%)	1 (1.3)	3 (4.2)	4 (3.6)	**5 (10.4)**	13 (4.2)
Census n (%)	1 (1.3)	2 (2.8)	1 (0.9)	5 (10.4)	9 (2.9)
National Center Health Statistics n (%)	3 (3.8)	1 (1.4)	3 (2.7)	1 (2.1)	8 (2.6)
CDC Wonder n (%)	1 (1.3)	0 (0.0)	1 (0.9)	1 (2.1)	3 (1.0)
BRFSS n (%)	0 (0.0)	1 (1.4)	0 (0.0)	0 (0.0)	1 (0.3)
NHANES n (%)	0 (0.0)	1 (1.4)	0 (0.0)	0 (0.0)	1 (0.3)
American Fact Finder n (%)	0 (0.0)	0 (0.0)	0 (0.0)	0 (0.0)	0 (0.0)
Dartmouth Atlas n (%)	0 (0.0)	0 (0.0)	0 (0.0)	0 (0.0)	0 (0.0)

Abbreviations: Agency for Healthcare Research and Quality (AHRQ); Behavioral Risk Factor Surveillance System (BRFSS); Centers for Disease Control and Prevention (CDC); confidence interval (CI); National Center Health Statistics (NCHS); National Health and Nutrition Examination Survey (NHANES);; odds ratio (OR); standard deviation (SD).Statistically significant values (*p* < 0.05) are in bold text, calculating using logistic regression analysis adjusted for terminal pharmacy degree, pharmacy residency, Board of Pharmacy Specialties certification, and population density.

**Table 6:** Association between Pharmacists’ Population-Based Information Source(s) Utilized and Continuing Education Topic Completion (n = 1279).

Continuing Education Topic	Information Source(s) Utilized			
	Local Health, Local Non-Health, and/or Associations ONLY		One or More Population-Based Information Resources	
	n (%)	Adjusted OR (95% CI) ^a^	n (%)	Adjusted OR (95% CI) ^a’^ ^459+^
Alzheimer disease	132 (22.5%)	1.00 (referent)	**220 (31.3%)**	**1.58 (1.21–2.05)**
Asthma	183 (31.2%)	1.00 (referent)	245 (34.9%)	1.25 (0.98-0.59)
Community/Population need	71 (12.1%)	1.00 (referent)	**39 (19.8%)**	**1.83 (1.34–2.51)**
Chronic obstructive pulmonary disease	157 (26.8%)	1.00 (referent)	**227 (32.3%)**	**1.30 (1.02–1.37)**
Dementia with Lewy bodies	35 (6.0%)	1.00 (referent)	63 (9.0%)	1.42 (0.92–2.21)
Diabetes	342 (58.3%)	1.00 (referent)	**467 (66.5%)**	**1.40 (1.11–1.76)**
Drug-induced cognitive problems	54 (9.2%)	1.00 (referent)	**106 (15.1%)**	**1.68 (1.18–2.40)**
Epilepsy	45 (7.7%)	1.00 (referent)	66 (9.4%)	1.27 (0.85–1.90)
Falls	74 (12.6%)	1.00 (referent)	**133 (19.0%)**	**1.55 (1.14–2.12)**
Geriatric syndrome	31 (5.3%)	1.00 (referent)	**63 (9.0%)**	**1.64 (1.04–2.57)**
Health disparities	44 (7.5%)	1.00 (referent)	**107 (15.2%)**	**2.16 (1.49–3.15)**
Heart failure	191 (32.5%)	1.00 (referent)	**278 (39.6%)**	**1.40 (1.10–1.77)**
Hypertension	220 (37.5%)	1.00 (referent)	**314 (44.7%)**	**1.37 (1.09–1.72)**
Incontinence	91 (15.5%)	1.00 (referent)	**156 (22.2%)**	**1.59 (1.19–2.15)**
Non–Alzheimer dementia	25 (4.3%)	1.00 (referent)	35 (5.0%)	1.07 (0.63–1.83)
Parkinson disease	88 (15.0%)	1.00 (referent)	**141 (20.1%)**	**1.45 (1.08–1.96)**
Parkinson disease dementia	28 (4.8%)	1.00 (referent)	52 (7.4%)	1.50 (0.92–2.45)
Physiology of aging	33 (5.6%)	1.00 (referent)	**62 (8.8%)**	**1.63 (1.03–2.57)**
Reversible dementia	12 (2.0%)	1.00 (referent)	22 (3.1%)	1.65 (0.79–3.46)
Transient ischemic attack	113 (19.3%)	1.00 (referent)	160 (22.8%)	1.24 (0.94–1.63)
Vascular dementia	15 (2.6%)	1.00 (referent)	31 (4.4%)	1.72 (0.91–3.23)

**^a^** Odds ratios and confidence intervals for logistic regression analysis adjusted for terminal pharmacy degree, pharmacy residency, Board of Pharmacy Specialties certification, and a primary workplace in academia or long–term care. Statistically significant values are in bold text.
